# Linezolid pharmacokinetics in critically ill patients with renal replacement therapy: comparison of equi-dose of continuous veno-venous haemofiltration with continuous veno-venous haemodiafiltration

**DOI:** 10.1186/2197-425X-3-S1-A631

**Published:** 2015-10-01

**Authors:** C Roger, B Louart, L Muller, JA Roberts, JY Lefrant

**Affiliations:** Nimes University Hospital, Nimes, France; University of Queensland, Brisbane, Australia

## Introduction

Linezolid is a commonly used antibiotic for difficult-to-treat Gram-positive infections for which little data is available to guide dosing for different types of renal replacement therapy.

## Objectives

The objective of this study was to compare the population pharmacokinetics of linezolid during continuous venovenous haemofiltration (CVVHF, 30 mL.kg^-1^.h^-1^) and continuous venovenous haemodiafiltration (CVVHDF, 15 mL.kg^-1^.h^-1^ + 15 mL.kg^-1^.h^-1^). We then sought to perform Monte Carlo dosing simulations to determine doses that best achieve pharmacodynamic targets for these patients.

## Methods

Patients with a clinical indication for linezolid and prescribed either CVVHF or CVVHDF were eligible for participation in this prospective pharmacokinetic study. Patients were administered 600 mg IV 12-hourly. Seven blood samples were collected over one dosing interval and analysed by a validated chromatographic method. Population pharmacokinetic analysis was undertaken using Pmetrics and Monte Carlo simulations evaluated achievement of a pharmacodynamics target of an area under the concentration-time curve from 0-24 hours to minimum inhibitory concentration (AUC_0-24_/MIC) of 80.

## Results

9 CVVHDF and 8 CVVHF were performed in 13 patients. Patient characteristics regimens of CVVHDF and CVVHF were similar. A two compartment linear model best described the data. CVVHDF was associated with a 20.5% higher mean linezolid clearance than CVVHF, without statistical significance (P = 0.39). Both increasing patient weight and decreasing SOFA score were associated with increasing linezolid clearance. The mean (SD) parameter estimates were clearance 3.8 (2.2) L/h, volume of the central compartment 26.5 (10.3) L, intercompartmental clearance constants from central to peripheral (Kcp) 8.1 (12.1) L/h and peripheral to central compartments (Kpc) 3.6 (4.0) L.h^-1^. Achievement of pharmacodynamics targets was low for a MIC of 2 mg/L with the studied dose.

## Conclusions

The present data indicates profound pharmacokinetic variability of linezolid during CVVHF and CVVHDF. Sub-optimal achievement of therapeutic targets occurs at the EUCAST breakpoint MIC of 2 mg/L using 600 mg IV 12-hourly.

**Figure 1:**
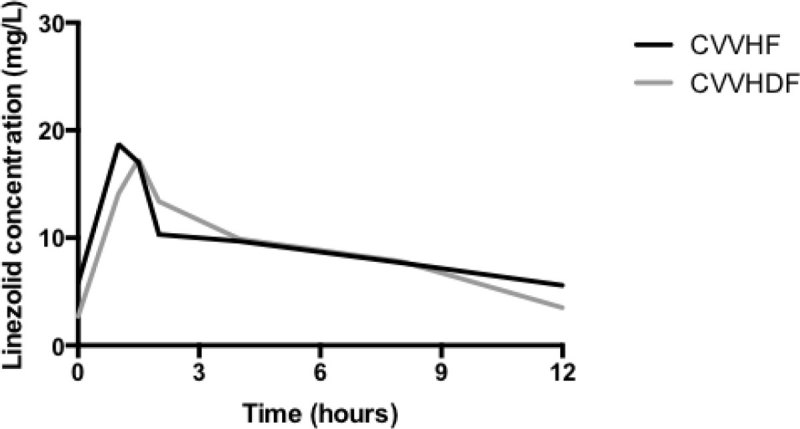
**The median observed linezolid concentration-time p.**

